# Problematic anger among military personnel after combat deployment: prevalence and risk factors

**DOI:** 10.1186/s40359-024-01955-8

**Published:** 2024-08-23

**Authors:** Andreas Espetvedt Nordstrand, Frederick Anyan, Hans Jakob Bøe, Odin Hjemdal, Laura Katherine Noll, Jon Gerhard Reichelt, David Forbes, Amy B. Adler

**Affiliations:** 1 Joint Medical Services, Institute of Military Psychiatry, Norwegian Armed Forces, Grev Wedels plass 2, Oslo, 0015, Norway; 2 Department of Psychology, Norwegian University of Science and Technology (NTNU), Trondheim, Norway; 3 Department of Psychological Sciences, Northern Arizona University (NAU), Flagstaff, AZ USA; 4 Department of Psychiatry, University of Melbourne, Melbourne, Australia; 5 Walter Reed Army Institute of Research, Silver Spring, MD USA; 6 Department of Psychology, University of Oslo (UiO), Oslo, Norway

**Keywords:** Anger, Military, Veterans, Mental health, Chronic pain, Transition

## Abstract

**Background:**

Problematic anger, characterized by excessive frequency, intensity, and duration of anger which causes substantial emotional distress and functional interference, poses a marked challenge in military populations. Despite its importance, research on this topic is limited. This study contributes to the literature by exploring problematic anger in a large sample of Norwegian military personnel who served in NATO missions in Afghanistan.

**Methods:**

All Norwegian military personnel who deployed to Afghanistan between 2001 and 2020 were sent a link to a cross-sectional web-based survey by the Joint Medical Services of the Norwegian Armed Forces in 2020. A total of 6205 individuals (response rate: 67.7%) participated. The cross-sectional survey assessed problematic anger, mental and physical health, war zone stressor exposure, and quality of life.

**Results:**

Overall, 8.4% of participants reported problematic anger. Mental health disorders, deployment-related shame and guilt, chronic pain, and challenges with the military-to-civilian transition were independently associated with problematic anger. Both staying in service and maintaining a part-time connection with the military as a reservist mitigated the risk of problematic anger after deployment, compared to complete separation from military service.

**Conclusion:**

Findings demonstrate a sizeable prevalence of problematic anger among veterans of combat deployments. Given the associations between problematic anger and mental health disorders, chronic pain, and transition challenges, interventions designed to mitigate problematic anger need to be multi-faceted, including the possibility of maintaining an ongoing connection to military service. By reducing the risk of problematic anger, occupational, interpersonal and health outcomes may be improved for service members. Future research should examine the impact of problematic anger on adjustment over time, prevention strategies, and problematic anger in other high-risk occupations.

**Supplementary Information:**

The online version contains supplementary material available at 10.1186/s40359-024-01955-8.

## Introduction

Anger is a ubiquitous emotion important for guiding adaptive human behavior [17] however, anger can be problematic when it is experienced with excessive frequency, intensity, and duration and disrupts daily functioning, relationships, and the emotional wellbeing [22]. Problematic anger also increases the risk of other mental health difficulties, such as insomnia, anxiety, depression, posttraumatic stress disorder (PTSD), and substance abuse [2, 23, 27]. Moreover, in the general population, inappropriate, intense, and poorly controlled anger has been associated with decreased psychosocial functioning [65], making it an important target for study in populations at elevated risk for problematic anger.

Disruptive levels of anger are increasingly recognized as a major concern after exposure to traumatic stressors [15, 38, 58]. Military service members deploying to a war zone are at risk of exposure to potentially traumatic stressors, such as physical danger, exposure to the death and suffering of others, and negotiating moral challenges [62]. And indeed, studies largely conducted with English-speaking samples have found that problematic anger is a common psychological challenge among military personnel and veterans returning from combat deployment [26, 27, 66]. Recent literature reviews have also highlighted the need to address problematic anger directly in treatment of trauma-related mental health problems [26, 58]. In part, this need is driven by findings that evidence-based treatments for PTSD are less effective for individuals with problematic anger [49] and that most military personnel still reported irritability and anger as residual symptoms after treatment [76].

Despite these findings, academic research on problematic anger among combat veterans is relatively nascent. Accordingly, Forbes and colleagues [26] have commented that “while problematic anger is a symptom or feature of multiple psychiatric disorders, this transdiagnostic lens has failed to compel adequate attention to the issue of anger in its own right”, and have labelled anger the “neglected emotion” [26, 27]. To address this gap, there have been calls for further assessment of the scope of problematic anger in military populations and exploration of potential associations with other areas of concern, particularly shame and guilt [58], chronic pain [52], and the military-to–civilian transition [3].

While there are many studies in forensic populations that have demonstrated the association between anger and emotions such as shame and guilt (e.g., [68, 84, 88]), few studies have examined this relationship in trauma-exposed populations. Both shame and guilt are common emotions after trauma exposure [1] in civilian [78] and military [48, 61, 91] populations. In addition, the level of trauma-related shame and guilt in the military context is often exacerbated by the unique moral and ethical challenges commonly encountered by soldiers serving in combat zones [74]. Accordingly, examining shame and guilt in terms of their association with problematic anger among combat veterans is likely to be informative.

Shame and guilt, although related, have distinct characteristics and implications for human behavior. Shame typically involves a painful, self-focused experience, eliciting feelings of worthlessness, powerlessness, and inferiority [28, 83]. Conversely, guilt typically arises from concern about a specific act and its potential harm leading to negative evaluation of the behavior, rather than the self [85]. Thus, it is important to consider these emotions as independent factors in post-deployment mental health [10, 69].

In the context of military trauma, the relationship between chronic pain and problematic anger is underexamined. Chronic pain is a commonly occurring health issue in military populations and is associated with combat exposure [32]. A recent systematic review found that chronic pain affects between 25 and 72% of all military veterans [8]. This estimate stands in contrast to prevalence estimates in the general population, where the same study found prevalence rates of 18–35% among civilians. Chronic pain has implications not only for quality of life but also for occupational functioning. For example, musculoskeletal pain is the primary reason for medical discharge among US service members [77]. While acute pain is caused by actual or potential tissue injury, less is known about the causes of chronic pain [14]. Leading theories of pain such as the gate control theory [56] and neuromatrix theory (e.g., [54]) maintain that psychological distress, such as problematic anger, can increase pain by altering descending and central pain modulation systems [30]. Despite the prevalence of chronic pain and its associations with combat exposure in military samples, few studies have examined how it relates to problematic anger in these populations.

The military-to-civilian transition may also provide insight into understanding problematic anger. For individuals transitioning from actively serving in the military to veteran status, the experience can be punctuated by challenges that exacerbate mental health difficulties [87, 59]. Moreover, the sudden alteration in structure and camaraderie, potential uncertainty regarding employment and living arrangements, as well as pre-existing psychological issues associated with military war zone service may contribute to the complexity of this transition [39]. Thus, it is not surprising that the transition to civilian life can render veterans more prone to frustration and negative emotions [42], which may increase the likelihood of developing problematic levels of anger. Many studies have examined emotional difficulties such as PTSD, depression and anxiety experienced by veterans after they enter civilian life (e.g., [87]. The relationship between military personnel entering the civilian world and the phenomenon of problematic anger is, however, relatively unexplored. In an exception, one study found significant associations between problematic anger during the transition and emotional, relational, and financial functioning approximately 5 years later [3]. Such findings suggest the importance of considering the military-to-civilian transition in modeling risk of problematic anger.

Currently, research investigating anger among service members and veterans has highlighted the prevalence, importance, and relevance of this topic. These studies have identified the prevalence of problematic anger as higher than the prevalence of mental health disorders such as PTSD and depression [90]. However, to date, there are still few studies on problematic anger in military populations, and the existing research is mostly limited to the English-speaking contexts [2, 90]. Accordingly, in the present study we aim to add to this emerging literature by investigating the scope of problematic anger in a large and highly trauma-exposed cohort of Norwegian service members and veterans who deployed to Afghanistan. The study also aims to assess the association between problematic anger and trauma-related shame and guilt, chronic pain, as well as military-to-civilian transition status, alongside more commonly evaluated markers of mental health (i.e., depression, PTSD, anxiety, insomnia, hazardous drinking, and satisfaction with life).

## Methods

### Study population

In 2020, the Joint Medical Services of the Norwegian Armed Forces conducted a large-scale study of all Norwegian military personnel who participated in the NATO missions in Afghanistan. This cross-sectional survey assessed war zone stressor exposure, mental and physical health, and quality of life (see supplemental file for all survey items used in the current study). All individuals who deployed to Afghanistan between 2001 and 2020 were invited to participate in a web-based survey (*N* = 9168). Of those invited, 145 (1.6%) declined participation and 2818 (30.7%) did not respond. In total, 6205 gave their consent to participate, resulting in a final response rate of 67.7%. Researchers only had access to anonymized data. Sample size variations due to missing demographic data and missing responses are reflected in the *n* values associated with specific analyses.

### Survey procedure

Information letters about the study were sent to all potential respondents, as well as a text message with a link to the web-based survey. A lottery of 30 computer tablets among participants was given as incentive to respond to the survey. The data collection took place between September 24th to November 24th, 2020, and included two reminders to non-responders. The participants gave their informed consent before responding to the survey. All procedures, data collection, storing, and distribution of data were made in accordance with the legislation regulating the Norwegian Armed Forces Health Registry. The study followed the Norwegian legislation for health research, the Helsinki declaration standards for ethical research, and was approved by the Regional Committee for Medical and Health Research Ethics (REK) of South-East Norway (case number: 33032).

### Demographics

Information concerning several demographic variables were extracted from the Norwegian Armed Forces personnel records, not through the survey. These variables include age, sex, and military status/rank. However, respondents reported their cohabitation status, highest educational attainment, and employment status on the survey.

### Measures

#### Outcome variable

Problematic anger was assessed using the 5-item Dimensions of Anger Reactions (DAR-5) scale [24]. The DAR-5 is designed to evaluate the frequency, intensity, and duration of anger, aggressive impulses, and the impact of anger on an individual’s social functioning, referencing the past four weeks of their experience. Each item (e.g., “When I got angry, I got really mad”) is rated on a 5-point Likert scale ranging from (1) “none or almost none of the time” to (5) “all or almost all of the time.” The sum of items resulted in a possible total score range from 5 to 25, with higher scores indicating higher levels of anger. A validated cut-off score of ≥ 12 on the DAR-5 was utilized to identify individuals with problematic anger [24]. The reliability of the DAR-5 in our study sample, as indicated by Cronbach’s alpha, was acceptable (*M* = 7.45, *SD* = 2.69, *α* = 0.79). The DAR-5 has been demonstrated to be psychometrically robust, and the established cut-off score has been confirmed in studies translating the measure (e.g., Arabic [40]; French [12]).

### Predictor variables

#### Military-to-civilian transition

Military-to-civilian transition status was assessed by two survey items. First, the question “Did you become a civilian or did you continue in the Armed Forces when you came home from your last deployment in Afghanistan?”, with the response options “Left the military and became a civilian immediately after returning from the deployment” or “Continued in military service after returning from the deployment”. Second, the question “What is your connection to the Armed Forces today?”, with the response options: “Full time employed in the Armed Forces”,” Civilian, no connection to the Armed Forces”, and “Civilian, but member of the Armed Forces reserve/home guard.” By creating an algorithm based on the response options of these two questions we were able to identify five transition-status groups in the study sample: no transition, returned to military service after transitioning to civilian, immediate transition to civilian after deployment, delayed transition to civilian, and transition to civilian with reserve/home guard service.

#### War zone stressors

War zone stressors were assessed using the war zone stressor exposure index (WarZEI), which capture a diverse range of deployment-related potentially traumatic events. The items and response options were adapted from measures of combat experiences used in military research (e.g., [36, 81, 86]) that have been previously validated (e.g., [33, 94]). The WarZEI has been previously used by the Norwegian Armed Forces in a range of studies (e.g., [62, 63, 75]). The index consists of 20 items (e.g., “I was attached by the enemy”, “I managed dead bodies or body parts” or “I participated in morally transgressive actions”). Each item was rated on a 5-point scale with the response options: 0 (not experienced), 1 (experienced 1–2 times), 2 (experienced 3–12 times), 3 (experienced 13–50 times), and 4 (experienced 50 + times). Individuals were instructed to respond to the items in terms of their military service in Afghanistan as whole. Items were summed, resulting in scores ranging from 0 to 50 (*n* = 6151, *M* = 9.7, *SD* = 7.8). Higher scores indicate a greater exposure load. Following the approach of Rønning and colleagues [75], four groups were created for analysis, corresponding to different levels of trauma exposure: (1) No exposure, (2) Low exposure, (3) Moderate exposure and (4) High exposure. The 4 groups were created by first identifying the respondents reporting no exposure, and then dividing the exposed participants into tertiles based on the reported frequency of trauma-exposure.

#### Chronic pain

Four items assessing chronic pain were sourced from the fourth wave of the large-scale Health Survey in Trøndelag (HUNT-4; e.g., [45]). These items were selected by the HUNT-4 project group based on several common self-report pain measures designed to map physical health complaints in population-sized samples. Symptoms of chronic pain in different organ systems were assessed by four questions. Muscle and joint pain were assessed by the question, “Have you been troubled by pain in muscles and joints continuously for at least 3 months during the last 12 months?” [46]; gastrointestinal complaints were assessed by the question, “Have you been troubled by persistent gastrointestinal pain or discomfort in the last 12 months?”  [18]; headache was assessed by the question, “Have you been troubled by persistent headaches in the 12 months?” [34]; and physical exhaustion/fatigue was assessed by the question, “Have you felt persistently exhausted/tired in the 12 months?” [92]. Response options for each item were “Yes” or “No.”

#### Psychological health

##### Posttraumatic stress disorder

Probable PTSD was assessed with the 20-item Post Traumatic Stress Disorder Checklist 5 (PCL-5; [93]). The PCL-5 aligns with the DSM-5 criteria for PTSD and examines symptoms such as reliving traumatic experiences, avoidance of trauma reminders, negative alterations in mood and cognition, and changes in arousal and reactivity [93]. Participants rated each symptom in terms of the past month on a 5-point Likert scale, ranging from 0 (“Not at all”) to 4 (“Extremely”), giving a possible total score range of 0 to 80, with higher scores representing greater severity of PTSD symptoms. A score on the PCL-5 between 31 and 33 is recommended as a cut-off score for probable PTSD across samples [95]. In this study, we used a cut-off score of 32. The internal consistency of the PCL-5 in the current sample was excellent (*M* = 5.91, *SD* = 9.56, *α* = 0.95).

##### Shame and guilt

Deployment-related shame (*M* = 0.59, *SD* = 1.06, *α* = 0.65) and deployment-related guilt (*M* = 0.47, *SD* = 1.23, *α* = 0.84), was assessed using the 9-item Shame and Guilt After Trauma Scale (SGATS; [1]). The SGATS measures both trauma-related guilt (five items; e.g., “Have you ever felt like you did something wrong during your deployment?”) and shame (four items; e.g., “Have you worried about what other people might think of you after the deployment?”). Items were rated in terms of agreement using three response options: 0 (no shame/guilt), 1 (yes, some shame/guilt), or 2 (yes, significant shame/guilt). In the current study, we followed the suggestion by the original authors [1], and dichotomized the respondent’s scores by collapsing responses of 1 and 2, giving a possible 0 or 1 score indicating the presence/absence of deployment related shame and guilt.

##### Anxiety and depression

Anxiety and depression was assessed using the Hospital Anxiety and Depression Scale (HADS; [96]. The scale contains 14 items and consists of two subscales: Anxiety (HADS-A; seven items) and Depression (HADS-D; seven items). The two subscales have been found to be sensitive, symptom-specific, and valid for independent use as separate indices of anxiety and depression [9]. Each item is rated on a 4-point severity scale ranging from 0 to 3. In the current study, we used the HADS-A and HADS-D to indicate the levels of anxiety (*M* = 3.39, *SD* = 3.23, *α* = 0.81) and depression (*M* = 2.51, *SD* = 3.09, *α* = 0.83) respectively. Items were summed, yielding a possible total score ranging from 0 to 21 for each of the two subscales. Higher scores indicate elevated symptom levels, and scores above 11 are considered indicative of clinical levels of depression or anxiety [96].

##### Insomnia

The 7-item Insomnia Severity Index (ISI; [7] was used to capture reports of insomnia in the study (*M* = 4.42, *SD* = 5.16, *α* = 0.87). Each item (e.g., “How satisfied/dissatisfied are you with your current sleep pattern?”) is rated on a 5-point scale that ranges from 0 to 4, giving a total score range of 0 to 28, with higher scores reflecting a higher level of symptoms. A tallied score of 15 or more has been suggested as indicating clinical sleep problems [60], and was used as the cut-off score for insomnia in the current study.

##### Hazardous drinking

The Alcohol Use Disorder Identification Test (AUDIT; [5] is a 10-item questionnaire developed by the World Health Organization to identify individuals with hazardous drinking patterns (*M* = 5.32, *SD* = 3.40, *α* = 0.72). Eight items regarding current alcohol use were rated on a 5-point scale from 0 to 4, and two items were rated with scores of 0, 2, or 4, giving a total possible score range of 0 to 40, with higher scores indicating more alcohol use. The recognized cut-off score of 16 [5] was used to indicate hazardous drinking in this study.

##### Satisfaction with life

We used the 5-item Satisfaction with Life Scale (SWLS; [19] to obtain an indication of quality of life/life satisfaction among the respondents. Items (e.g., “In most ways, my life is the way I want it to be.”) are rated on a scale from (1) “Strongly disagree” to (5) “Strongly agree.” In this sample, the instrument showed excellent internal consistency (*M* = 28.04, *SD* = 6.05, *α* = 0.92). Items were summed and levels of life satisfaction were determined using norms suggested by a Norwegian SWLS validation study [13]: Slightly to extremely dissatisfied = 5–19, Average satisfaction = 20–24, Satisfied to extremely satisfied = 25–35.

### Statistical analyses

Descriptive analyses were first performed followed by bivariate logistic regression analyses to assess the relationship between the different categories of variables and problematic anger. Categories of factors were, *(i) demographic factors*: age, sex, cohabitation, education, and employment status; (ii) *military service background characteristics*: military rank, transition status, and war zone exposure; *ii) physical health*: head, gastrointestinal, fatigue, and muscle pains; *(iii) psychological health*: deployment related shame, and guilt, posttraumatic stress disorder, insomnia, anxiety, depression, and hazardous drinking, and satisfaction with life.

We computed two multivariate logistic regression models. The first was an unadjusted model which did not control for the effects of other variables. The second was a fully adjusted model that accounted for the unique effects of each variable above and beyond other variables, thus determining which factors were independently associated with problematic anger.

To assess how many percentage points of problematic anger could be reduced in this population if certain factors were eliminated (e.g., probable PTSD), the population attributable risk percent (PAR%) was calculated as the prevalence of the factor among those with problematic anger multiplied by the adjusted odds ratio (AOR) minus 1 divided by the OR multiplied by 100 [(i.e., prevalence among cases × [(OR − 1)/OR] × 100%)]. All analyses were performed using Stata 14 [80].

### Data preparation and missing data

The data were screened for multicollinearity. Inspection of the correlation matrix showed that correlations were generally low to modest (all < 0.50). The variance inflation factor (VIF) ranged between 1.06 and 1.74, far below the cutoff (10.0) and the tolerance statistic was all above the cut-off (0.10), ranging between 0.57 and 0.94. There were no missing data reported on age and sex. Except measures of PTSD (9.65%), hazardous drinking (7.45%), and military-to-civilian transition status (5.12%), remaining model predictors as well as problematic anger had less than 5% missing data. Little’s test of missing completely at random (MCAR) failed to reject the null hypothesis of MCAR [47]. Multiple imputation using iterative chained equations (MICE) with 20 imputed datasets was used to retain participants.

## Results

### Descriptives

Table 1 provides demographic information on the sample. The study participants were predominantly males (91.7%). Ages ranged from 20 to 80 years old, with a mean age of 42. The individuals with age close to 80 years old at survey time represent subject matter experts, (i.e., explosive disarmament experts), brought back from retirement in the early years of Norway’s engagement in Afghanistan. About 80% of the entire sample were cohabiting with a spouse or partner. In terms of education, about 36% had completed a bachelor’s degree while 35% had completed postgraduate education. In terms of employment, 87.5% were full-time employees. The most common military rank was junior officer (38.5%); and 2.0% were deployed as civilians (in Norway, civilians who deployed with the Armed Forces wear military uniforms, are armed, and also regarded as veterans after deployment). At the time of the survey, 43% of the full sample was still on active duty.

**Table 1:** Frequencies and percentage of participant characteristics by problematic anger status

Variables		Study sample (*N*)	No problematic anger		Problematic anger	
			*n*	%	*n*	%
Sample		6205	5428	91.6%	496	8.4%
**Demographics**						
Age						
Below 42 years		3499	2951	54.4%	352	71.0%
42 years or more		2706	2477	45.6%	144	29.0%
Sex						
Female		512	455	8.4%	38	7.7%
Male		5693	4973	91.6%	458	92.3%
Cohabitation status						
No		1237	1061	19.5%	109	22.0%
Yes		4959	4367	80.5%	387	78.0%
Highest civilian education						
Elementary school		72	57	1.1%	9	1.8%
High school		886	171	13.7%	31	21.8%
Vocational education		829	570	12.9%	77	16.1%
Bachelor’s degree		2232	1987	36.6%	165	33.3%
Postgraduate degree		2174	1944	35.8%	134	27.0%
Employment status						
Fulltime employee		5421	4784	88.1%	408	82.3%
Part-time employee		72	57	1.1%	11	2.2%
Self-employed		161	131	2.4%	19	3.8%
Unemployed, receiving benefits		163	113	2.1%	38	7.7%
Retired		280	264	4.9%	8	1.6%
Other		97	79	1.5%	12	2.4%
**Military service background characteristics**						
Rank						
Junior Enlisted		1662	1345	25.3%	212	43.7%
Non-commissioned Officer		589	512	9.6%	49	10.1%
Junior officer		2391	2118	39.9%	171	35.3%
Senior officer		1313	1235	23.2%	42	8.7%
Civilian		120	104	2.0%	11	2.3%
Transition status						
No transition		2500	2309	44.7%	120	25.7%
Returned to military service		189	157	3.0%	24	5.1%
Immediate transition to civilian		1435	1185	22.9%	154	33.0%
Delayed transition to civilian		1280	1106	21.4%	120	25.7%
Transition to civilian with reserve/home guard service		483	708	7.9%	49	10.5%
War zone exposure						
No exposure		450	417	7.7%	18	3.6%
Low exposure		2141	1968	36.3%	97	19.6%
Moderate exposure		1742	1523	28.1%	146	29.4%
High exposure		1824	1520	28.0%	235	47.4%
**Physical health**						
Headaches						
No		4383	4125	76.0%	238	48.0%
Yes		1569	1303	24.0%	258	52.0%
Gastrointestinal pain						
No		4021	3786	69.7%	221	44.6%
Yes		1927	1448	30.3%	207	55.4%
Fatigue						
No		4680	4453	82.0%	211	42.5%
Yes		1267	975	18.0%	285	57.5%
Muscle and joint pain						
No		4266	3993	73.6%	257	51.8%
Yes		1680	1435	26.4%	239	48.2%
**Psychological health**						
Deployment-related shame						
No		3927	3710	68.3%	196	39.5%
Yes		2032	1718	31.7%	300	60.5%
Deployment-related guilt						
No		4781	4480	82.5%	274	55.2%
Yes		1178	948	17.5%	222	44.8%
Posttraumatic stress disorder						
No		5426	4942	98.6%	372	77.8%
Yes		180	69	1.4%	106	22.2%
Insomnia						
No		5636	5247	96.7%	377	76.0%
Yes		300	181	3.3%	119	24.0%
Anxiety						
No		5693	5312	97.9%	380	76.6%
Yes		233	116	2.1%	116	23.4%
Depression						
No		5761	5345	98.5%	414	83.5%
Yes		165	83	1.5%	82	16.5%
Hazardous drinking						
No		5645	5217	98.9%	427	91.8%
Yes		98	60	1.1%	38	8.2%
Satisfaction with life						
Slightly to extremely dissatisfied		643	458	8.5%	185	37.6%
Average satisfaction		596	501	9.3%	95	19.3%
Satisfied to extremely satisfied		4668	4456	82.3%	212	43.1%

**Fig. 1:**
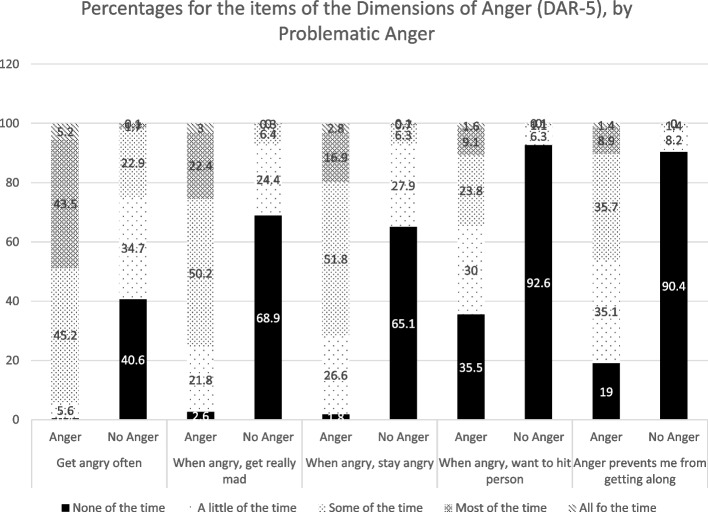
Percentages for the items of the Dimensions of Anger (DAR-5), by Problematic Anger. i. I often find myself getting angry at people or situation. ii. When I get angry, I get mad. iii. When I get angry, I stay angry. iv. When I get angry at someone, I want to hit or clobber the person. v. My anger prevents me from getting along with people as well as I’d like to

Table 1 presents characteristics and frequencies by problematic anger. Overall, 496 (8.4%) had problematic anger. We followed the previous study by Adler et al. [2] to compute endorsement of problematic anger by using the responses from “*Some of the time”* to “*All of the time*”. Across participants, 29.1% endorsed *“I often find myself getting angry at people or situations”*, 11.9% endorsed “*When I get angry*,* I get really mad*”, 11.9% endorsed *“When I get angry*,* I stay angry*”, 3.7% endorsed “*When I get angry at someone*,* I want to hit or clobber the person*”, and 4.9% endorsed “*My anger prevents me from getting along with people as well as I’d like to*”. Responses to the problematic anger items by problematic anger status are presented in Fig. 1.

### Predictors of problematic anger

Table 2 provides results for unadjusted and fully adjusted odd ratios for problematic anger. In the fully adjusted model, factors found to *decrease* the odds of problematic anger were higher age [AOR = 0.64; (95% CI: 0.48, 0.84)] compared to lower age (below 42 years), unemployed receiving benefits [AOR = 0.45; (95% CI: 0.25, 0.79)] compared to being in fulltime employment, and factors related to psychological wellbeing, including being satisfied to extremely satisfied with life [AOR = 0.33; (95% CI: 0.24, 0.46)] compared to low satisfaction. Other factors in the fully adjusted model were found to *increase* the odds of problematic anger. Cohabitating [AOR = 1.71; (95% CI: 1.29, 2.26) PAR% = 32.38] was more likely to be related to problematic anger than not living with a partner. Returning to military service after leaving the military [AOR = 2.61; (95% CI: 1.54, 4.43) PAR% = 3.15] and delayed transition [AOR = 1.47; (95% CI: 1.07, 2.02) PAR% = 8.22] were associated with greater likelihood of reporting problematic anger compared to staying in the military. Physical health problems related to headaches [AOR = 1.33; (95% CI: 1.06, 1.68) PAR% = 12.90], fatigue [AOR = 1.80; (95% CI: 1.40, 2.33) PAR% = 25.56], and muscle and joint pains [AOR = 1.39; (95% CI: 1.09, 1.79) PAR% = 13.52] were significantly associated with greater risk of problematic anger than those without symptoms of chronic pain. In terms of psychological health factors, deployment-related shame [AOR = 1.33; (95% CI: 1.05, 1.68), PAR% = 15.01] and guilt [AOR = 1.39; (95% CI: 1.09, 1.79), PAR% = 12.57] were associated with significantly greater risk of problematic anger than feeling no deployment-related shame and guilt. Similarly, clinical levels of PTSD symptoms [AOR = 2.66; (95% CI: 1.72, 4.12), PAR% = 13.85], insomnia [AOR = 1.63; (95% CI: 1.13, 2.34), PAR% = 9.28], anxiety [AOR = 1.90; (95% CI: 1.29, 2.78), PAR% = 11.08], and hazardous drinking [AOR = 1.86; (95% CI: 1.05, 3.30), PAR% = 3.79] were significantly more likely to be associated with problematic anger than normal levels.

**Table 2:** Relationship between predictor variables and problematic anger in unadjusted and fully adjusted models

**Variables**		Unadjusted model		Fully adjusted model	
		OR (95% CI)	*p*-value	AOR (95% CI)	*p*-value
Sample					
**Demographics**					
Age (ref: Below 42 years)					
42 years or more		0.49 (0.39, 0.59)	0.000	0.64 (0.48, 0.84)	0.001
Sex (ref: Female)					
Male		1.10 (0.78, 1.56)	0.575	0.92 (0.62, 1.34)	0.680
Cohabitation status (ref: No)					
Yes		0.85 (0.68, 1.05)	0.138	1.71 (1.29, 2.26)	0.000
Civilian education (ref: Elementary school)					
High school		0.88 (0.42, 1.84)	0.742	1.60 (0.65, 3.95)	0.303
Vocational education		0.68 (0.32, 1.44)	0.313	1.29 (0.52, 3.20)	0.587
Bachelor’s degree		0.49 (0.24, 1.03)	0.060	1.25 (0.52, 3.04)	0.618
Postgraduate degree		0.41 (0.19, 0.85)	0.017	1.29 (0.53, 3.15)	0.580
Employment status (ref: Fulltime employee)					
Part-time employee		2.46 (1.33, 4.80)	0.006	1.19 (0.52, 2.73)	0.673
Self-employed		1.68 (1.04, 2.72)	0.037	1.05 (0.59, 1.87)	0.854
Unemployed, receiving benefits		3.91 (2.70, 5.75)	0.000	0.45 (0.25, 0.79)	0.006
Retired		0.36 (0.17, 0.70)	0.005	0.72 (0.32, 1.63)	0.438
Other		1.92 (1.04, 3.52)	0.031	1.21 (0.59, 2.46)	0.596
**Military service background characteristics**					
Military rank/civilian service (ref: Junior enlisted)					
Non-commissioned Officer		0.60 (0.43, 0.83)	0.002	0.79 (0.54, 1.16)	0.246
Junior officer		0.50 (0.41, 0.62)	0.000	0.84 (0.63, 1.12)	0.233
Senior officer		0.21 (0.15, 0.30)	0.000	0.62 (0.38, 1.01)	0.055
Civilian		0.66 (0.34, 1.26)	0.209	1.07 (0.51, 2.26)	0.852
Transition status (ref: No transition)*					
Returned to military service		3.01 (1.88, 4.83)	0.000	2.61 (1.54, 4.43)	0.000
Immediate transition to civilian		2.52 (1.97, 3.22)	0.000	1.32 (0.97, 1.81)	0.081
Delayed transition to civilian		2.21 (1.69, 2.87)	0.000	1.47 (1.07, 2.02)	0.016
Transition to civilian with reserve/home guard service		2.31 (1.62, 3.29)	0.000	1.12 (0.72, 1.73)	0.617
War zone stressor exposure (ref: No exposure)					
Low exposure		1.17 (0.70, 1.96)	0.541	0.84 (0.49, 1.43)	0.518
Moderate exposure		2.31 (1.40, 3.81)	0.001	1.07 (0.62, 1.83)	0.799
High exposure		3.71 (2.28, 6.06)	0.000	1.15 (0.67, 1.97)	0.602
**Chronic Pain**					
Headaches (ref: No)					
Yes		3.50 (2.91, 4.21)	0.000	1.33 (1.06, 1.68)	0.014
Gastrointestinal pain (ref: No)					
Yes		2.91 (2.41, 3.52)	0.000	1.13 (0.89, 1.43)	0.300
Fatigue (ref: No)					
Yes		6.30 (5.21, 7.62)	0.000	1.80 (1.40, 2.33)	0.000
Muscle and joint pain (ref: No)					
Yes		2.61 (2.16, 3.14)	0.000	1.38 (1.10, 1.73)	0.005
**Psychological health**					
Deployment-related shame (ref: No)					
Yes		3.36 (2.78, 4.06)	0.000	1.33 (1.05, 1.68)	0.019
Deployment-related guilt (ref: No)					
Yes		3.85 (3.19, 4.66)	0.000	1.39 (1.09, 1.79)	0.009
Posttraumatic stress disorder (ref: No)					
Yes		21.39 (15.67, 29.21)	0.000	2.66 (1.72, 4.12)	0.000
Insomnia (ref: No)					
Yes		9.33 (7.26, 11.98)	0.000	1.63 (1.13, 2.34)	0.009
Anxiety (ref: No)					
Yes		14.15 (10.74, 18.64)	0.000	1.90 (1.29, 2.78)	0.001
Depression (ref: No)					
Yes		13.20 (9.56, 18.23)	0.000	1.44 (0.92, 2.26)	0.106
Problem drinking (ref: No)					
Yes		7.88 (5.15, 12.04)	0.000	1.86 (1.05, 3.30)	0.033
Satisfaction with life (ref: Slightly to extremely satisfied)					
Average satisfaction		0.46 (0.35, 0.61)	0.000	0.79 (0.57, 1.12)	0.191
Satisfied to extremely satisfied		0.11 (0.09, 0.14)	0.000	0.33 (0.24, 0.46)	0.000

*Immediate and delayed transition indicates respectively leaving military service upon arrival home from deployment or leaving military service at a later stage and currently civilian

## Discussion

The current study represents one of the first investigations of problematic anger among military personnel examining the links between problematic anger and central post-deployment topics such as shame, guilt, chronic pain, and military to civilian transition. Findings revealed that eight and a half percent of Norwegian service members and veterans who served in Afghanistan reported problematic anger, as measured by the DAR-5. Other mental health concerns (e.g., PTSD, depression, anxiety, hazardous drinking, insomnia) in the current sample are reported at around half this prevalence or less [11, 37]. The fact that problematic anger was the most commonly endorsed mental health concern is congruent with patterns found in previous studies (e.g., [2, 90]). However, despite the frequency with which military populations reports problematic anger, the phenomenon typically does not receive the same level of attention as more traditionally recognized clinical disorders [26]. Considering the current findings, problematic anger warrants increased attention in future health studies among military personnel.

By focusing on factors associated with problematic anger, researchers and clinicians may be able to develop approaches that better mitigate the risk for adverse outcomes following combat deployments. To that end, study results showed that even after adjusting for demographics, military service background, and other mental health disorders, new factors were identified as determinants of problematic anger. Specifically, in the fully adjusted model, deployment-related shame and guilt, headaches, fatigue, muscle and joint pain, and military-to-civilian transition status remained independently associated with problematic anger among soldiers who served in Afghanistan. While the study was cross-sectional and cannot address questions of directionality, the findings may be useful in informing intervention strategies that target risk factors (like pain or guilt) associated with problematic anger [26] or that target problematic anger directly (like addressing anger specifically when treating trauma-related mental health problems [58]).

Since research on problematic anger among military personnel is still nascent, discrepancies and similarities between the results in the present study and previous research are important to note. Consistent with previous results [2, 26], younger age was a predictor of problematic anger, although in our sample biological sex was not. Furthermore, in contrast to previous studies [2, 26], we found that neither military rank nor civilian education were associated with problematic anger. Such non-significant relationships associated with social status may seem counterintuitive but could reflect the relatively egalitarian nature of Norwegian society [35, 64], where differences in rank and education may not be as impactful in terms of self-concept and self-efficacy.

These societal conditions may also partially account for the unexpected finding that being unemployed with state benefits was associated with less risk of reporting problematic anger than full-time employment. Considering the fact that Norway provides liberal welfare benefits, the economic stress of being unemployed may be mitigated compared to what would be expected in other countries. Not working could also entail fewer social demands, which has been shown to result in less opportunities for anger reactions to be triggered [41]. This interpretation can also account for the association between problematic anger and cohabitating with someone, as observed in the current study. However, being unemployed and socially withdrawn is simultaneously known to increase the risk of other types of psychological distress such as depression, anxiety, and loneliness (e.g., [4]). Future research is needed to clarify these relationships but since social support and meaningful close relationships are important buffers of post-deployment mental health challenges (e.g. [63, 64]), social isolation likely does not represent a healthy anger management strategy.

Consistent with past findings (e.g., [2, 57, 82], the current study demonstrated that probable PTSD, depression, anxiety, insomnia, and hazardous drinking were all significantly and uniquely correlated with problematic anger. In addition, and consistent with previous results [25], we found that low life satisfaction was associated with problematic levels of anger. Collectively, these findings suggest that an association between a range of comorbid mental health disorders and problematic anger is the norm rather than the exception among service members and veterans following deployment. That is, individuals who have difficulties in one arena of mental health are likely to simultaneously struggle with problematic anger.

The present study also offers new contributions by documenting significant links between problematic anger and deployment-related shame and guilt. Both deployment-related shame and guilt were independent predictors of problematic anger, supporting the approach of assessing them as distinct emotional experiences in trauma exposed cohorts. Shame and guilt are traditionally regarded as emotions that can instigate defensive affective reactions in the form of anger expressions (e.g., [84]), but the interplay between these emotions and anger is likely complex [16]. As an example, both deployment-related shame and guilt may intensify anger as individuals struggle with feelings of powerlessness, resentment, and low self-worth, and then use anger to mask these vulnerabilities (e.g., [84, 97]). While the current results do not examine these mechanisms, future research should acknowledge the broader emotional context within which problematic anger may develop [6], and treat these emotions separately when modeling their possible role in problematic anger.

Another novel finding revealed by our study is the close link between chronic pain and problematic anger among the respondents. Chronic pain, a common health concern among service members and veterans [8], appears intricately linked with disruptive levels of anger. In our specific study, gastrointestinal chronic pain was not correlated with problematic anger in the fully adjusted model, but the other three metrics of chronic pain (i.e., muscle and joint pain, headaches, and fatigue) were. The aetiology of chronic pain is not well understood but it has been shown to precipitate and exacerbate trauma-related mental health problems [43, 89]. Accordingly, the relationship between problematic anger and chronic pain can be understood through the lens of both physiological and psychological perspectives. Physiologically, chronic pain can lead to a constant state of discomfort and stress, which may lower an individual’s threshold for frustration and anger [30]. Psychologically, the persistent nature of pain often leads to feelings of helplessness, loss of control, and a diminished quality of life, all of which can contribute to increased irritability and anger [67]. Furthermore, our results suggest that this anger is not merely a reaction to physical discomfort but is intertwined with the emotional responses to the pain and its impact on a veteran’s life. This finding aligns with modern theories of pain like the neuro matrix theory, which posits that psychological factors interact with pain perception [55], which in cases of persistent negative affect, will likely exacerbate anger responses [71].

In a similar vein, our results may offer support for hypotheses positing physical pain to be the somatic expression of trauma-related mental health disorders [79]. Anger is not only a conscious experience, but a state of physiological activation expressed in almost all organ systems [70]. As such, it could be that a portion of the respondents with problematic anger are experiencing a vicious circle of chronic pain symptoms and anger-related elevation of bodily arousal locked in bi-directional aggravation. By integrating pain management and psychological support, interventions may be able to address this complex interplay more holistically [50].

Finally, the process of transitioning from military to civilian life is a significant and potentially vulnerable phase in the lives of service members, and the current findings offer insights into the significant role problematic anger may play during this period. First, individuals who leave active military service following a combat deployment are at increased risk of problematic anger in contrast to those who remain in the service either on active duty or as reservists. Transitioning to a civilian life is often complex and challenging for soldiers [59], marked by significant adjustments in lifestyle, identity, and social networks [3]. Ex-servicemembers may struggle with the loss of the military’s structured environment, camaraderie, and sense of purpose [44, 51], all of which can be contributors to the heightened feelings of anger among this segment of our sample. Furthermore, navigating a new civilian life can involve economic challenges and work-related struggle. Previous work has shown significant associations between problematic anger and economic dysfunction [3], thus the increased economic instability associated with changing career may partially account for the increased levels of anger among veterans in our study. Taken together, these findings suggest taking a nuanced approach to supporting veterans departing military service. Concerted efforts to facilitate a smooth transition by helping veterans find sources of purpose and community in civilian life may reduce their risk of post-deployment problematic anger.

Interestingly, our results indicated that those who continue to serve part-time in the Armed Forces’ home guard (roughly equivalent to US Armed Forces reserve component) exhibit low levels of problematic anger, comparable to those who never left the military. This finding is particularly intriguing as it suggests that maintaining a connection with the military, even in a limited capacity, provides a buffer against the development of problematic anger in a way that full separation from military life does not. On a personal level, this buffering effect could be due to a continued sense of identity, purpose and meaning commonly offered by military service [42]. On a relational level, maintaining contact with a familiar military culture and community, with its associated social support networks, may also mitigate transition challenges [29, 31] and reduce the risk of problematic anger. The current results illustrate the value of policies that encourage part-time military engagement for those transitioning out of active duty. Such efforts may not only serve the military organization but seem to benefit the individuals as well. Of note though, our findings suggest that once an individual has left the service, there does not appear to be added value in returning to the military in terms of problematic anger.

Besides identifying determinants of risk, the present study did not find an association between amount of deployment-related trauma exposure and problematic anger in the fully adjusted model. While there is a well-documented association between traumatic experiences during military war zone deployments and mental health (e.g. [38, 62]), the lack of relationship observed here is likely due to the broad range of mental health disorders measured in the study, which together account for a substantial proportion of the variance in our model. However, this interpretation should be approached with caution until appropriate tests of longitudinal mediation models are conducted within experimental designs.

### Limitations

The present study had several limitations that warrant consideration. One limitation is that the cross-sectional design precludes the establishment of causality, leaving open the question of the temporal sequence of observed relationships. For example, it could be that health-related variables predict problematic anger, that problematic anger predicts health-related outcomes, or that a third variable predicts both health-related variables and problematic anger. Another limitation is reliance on retrospective self-report data, which may introduce potential recall bias and subjective interpretation issues [53]. This can have affected the accuracy of the reported experiences. Moreover, it is possible that missing data on some predictor variables may introduce bias into the findings; however, the missing data was MCAR and our analytical approach is expected to perform well with the levels of missing data in the current study [20, 21]. Furthermore, the specific war zone stressor exposure index used in the present study has not been validated, potentially introducing measurement error.

Finally, the sample was not balanced in terms of sex, and only 8% of the respondents were women. This imbalance suggests that the findings may not reflect the experience of women, and thus have limited generalizability. Future research should aim for a more balanced sex representation to ensure that findings are applicable to both men and women.

### Implications and further directions

This study’s findings have a number of implications for both clinical practice and future research. First, problematic anger emerged as the most prevalent marker of mental health difficulties in this population of Norwegian veterans and service member who fought in Afghanistan. Given that problematic anger is seldom assessed in veteran studies, likely due to not being an established clinical diagnosis, a substantial proportion of post-deployment difficulties may be overlooked. Thus, it is important to include assessments of problematic anger in future epidemiological and intervention efforts.

Second, from a clinical perspective, the associations between problematic anger and factors like deployment-related shame and guilt, chronic pain, and the military-to-civilian transition underscore the need for holistic clinical care approaches. These approaches should not only address problematic anger as an isolated symptom but consider the broader emotional and physical health context of veterans. Tailoring interventions to address these interconnected issues could lead to more effective prevention and treatment strategies, ultimately enhancing the well-being of both active-duty military personnel and veterans.

In addition, the present study points to several avenues for future research. For example, given that the data in the present study did not include details on whether individuals wanted to leave military service, it may be useful for future research to examine the relationship between the nature of the separation from military service and problematic anger. Likewise, investigating the longitudinal dynamics of problematic anger and its associated factors may be valuable in understanding the causal pathways and the progression of anger-related issues over time. This can also inform prevention efforts. Furthermore, exploring these relationships within other high-risk occupational groups, such as law enforcement, paramedics, and fire and rescue personnel, would enhance the generalizability and applicability of the current findings. Such populations have all been shown to demonstrate strong parallels to combat veterans in terms of stressor exposure and subsequent psychological distress (e.g., [72, 73]), and an exploration of problematic anger in these cohorts would address a critical knowledge gap. It may also be useful to examine how information about the overlap between chronic pain and problematic anger can inform both assessments and treatment conducted by healthcare providers. Additionally, research into the development and efficacy of interventions that specifically target problematic anger is crucial. Intervention evaluation studies are often resource intensive but could significantly advance the field of military mental health [58]. Given the substantial prevalence of problematic anger, now demonstrated in several large military cohorts across cultures, such studies would address a critical gap in treatment and rehabilitation efforts aimed at improving the lives of recent era combat veterans.

### Supplementary Information

Supplementary Material 1.

## Data Availability

The data that support the findings of this study are available upon formal requests directed to the Joint Medical Services’ military health registry at fsan.info@mil.no. The data are not publicly available due to data containing information that could compromise the privacy of research participants.

## Data availability

Data availability statement: The data that support the findings of this study are not publicly available due to the laws regulating military health data in the Norwegian Armed Forces, but can be accessed through reasonable request adressed to Norwegian Armed Forces Health Registry at datatilgang@forsvaretshelseregister.no.
