# Supplementary material for: Problematic anger among military personnel after combat deployment: prevalence and risk factors
Source: BMC Psychol. 2024 Aug 23;12:451. doi: 10.1186/s40359-024-01955-8 (PMC11344415; doi:10.1186/s40359-024-01955-8)
Supplement: Supplementary file 1 — Supplementary Material 1. [file 40359_2024_1955_MOESM1_ESM.docx]

**Supplemental Material**

**Survey Items Used in the Main Manuscript**

**Problematic Anger among Military Personnel after Combat Deployment: Prevalence and Risk Factors**

**Military to civilian transition items and response options**

- *Did you become a civilian or did you continue in the Armed Forces when you came home from Afghanistan?* Response options: 1. “Left the military and became a civilian immediately after returning from the deployment” or 2. “Continued in military service after returning from the deployment”.
- *What is your connection to the Armed Forces today?* Response options: 1. “Full time employed in the Armed Forces”, 2.” Civilian, no connection to the Armed Forces”, and 3. “Civilian, but member of the Armed Forces reserve/home guard.”

The items were developed for the Afghanistan 2020 Veteran Survey. Items were translated from Norwegian.

**War zone stressor index items and response options**

- *I shot, directed, or led fire at the enemy.*
- *I have, or think I have, taken a life in the service.*
- *I was attacked (e.g., shelled, IED, suicide bombs, friendly fire).*
- *I was almost killed / seriously injured (“close call”).*
- *I witnessed the moment when someone was seriously injured or killed.*
- *I had a moment where I thought I was going to die.*
- *I operated in areas of high threat level (e.g., IED, hostile activity).*
- *I was in a very threatening situation (e.g., being threatened with a gun, surrounded by an angry mob).*
- *I saw seriously injured or dead people.*
- *I saw seriously ill, injured, or dead children.*
- *I looked after or otherwise handled dead bodies or body-parts.*
- *I felt the suffering and distress of the civilian population close to me.*
- *Someone close to me was seriously injured in the service.*
- *Someone close to me lost their life in the line of service.*
- *I witnessed something that was morally questionable.*
- *I was involved in something that was morally questionable.*
- *I failed to do something that I see in retrospect that I morally should have.*
- *I was in a serious accident (e.g. fire, car accident, breakdown).*
- *I was captured or taken over by the enemy (e.g.: carjacking, kidnapped, held hostage).*
- *I was physically injured in combat.*

Each item was rated on a 5-point scale: 0. (not experienced), 1. (experienced 1–2 times), 2. (experienced 3–12 times), 3. (experienced 13–50 times), and 4. (experienced 50+ times).

The items were developed for the Afghanistan 2020 Veteran Survey. Items were translated from Norwegian.

**Chronic Pain items and response options (Kuorinka et al., 1987; DeVault & Castell, 2005; Hagen et al., 2000; Ware et al., 2001)**

- *Have you been troubled by pain in muscles and joints continuously for at least 3 months during the last 12 months?*
- *Have you been troubled by persistent gastrointestinal pain or discomfort in the last 12 months?*
- *Have you been troubled by persistent headaches in the 12 months?*
- *Have you felt persistently exhausted/tired in the 12 months?*

Response options for each item were "Yes" or "No."

The items were developed for assessing the prevalence of chronic pain the Trøndelag Health Study (The HUNT Study), one of the largest health studies ever conducted. Items were translated from Norwegian.

**Problematic Anger (Dimensions of Anger Reactions [DAR-5]) items and response options (Forbes et al., 2014)**

- *I found myself getting angry at people or situations.*
- *When I got angry, I got really mad.*
- *When I got angry, I stayed angry.*
- *When I got angry at someone I wanted to hit them.*
- *My anger prevented me from getting along with people as well as I’d have liked to.*

Each item is rated on a 5-point scale in terms of the past 4 weeks: 1. None or almost none of the time, 2. A little of the time, 3. Some of the time, 4. Most of the time, 5. All or almost all of the time.

**Posttraumatic stress disorder (Posttraumatic check list [PCL-5], Weathers et al., 2013) items and response options**

- *Repeated, disturbing, and unwanted memories of the stressful experience?*
- *Repeated, disturbing dreams of the stressful experience?*
- *Suddenly feeling or acting as if the stressful experience were actually happening again (as if you were actually back there reliving it)?*
- *Feeling very upset when something reminded you of the stressful experience?*
- *Having strong physical reactions when something reminded you of the stressful experience (for example, heart pounding, trouble breathing, sweating)?*
- *Avoiding memories, thoughts, or feelings related to the stressful experience?*
- *Avoiding external reminders of the stressful experience (for example, people, places, conversations, activities, objects, or situations)?*
- *Trouble remembering important parts of the stressful experience?*
- *Having strong negative beliefs about yourself, other people, or the world (for example, having thoughts such as: I am bad, there is something seriously wrong with me, no one can be trusted, the world is completely dangerous)?*
- *Blaming yourself or someone else for the stressful experience or what happened after it?*
- *Having strong negative feelings such as fear, horror, anger,*
- *guilt, or shame?*
- *Loss of interest in activities that you used to enjoy?*
- *Feeling distant or cut off from other people?*
- *Trouble experiencing positive feelings (for example, being unable to feel happiness or have loving feelings for people close to you)?*
- *Irritable behavior, angry outbursts, or acting aggressively?*
- *Taking too many risks or doing things that could cause you harm?*
- *Being “superalert” or watchful or on guard?*
- *Feeling jumpy or easily startled?*
- *Having difficulty concentrating?*
- *Trouble falling or staying asleep?*

Each item is rated on a 5-point scale in terms of the past month: 1. Not at all, 2. A little bit, 3. Moderately, 4. Quite a bit, 5. Extremely. Items are rated on a scale from 0 to 4 with reference to the last two weeks; 0 represents low problem levels and 4 represents high problem levels.

**Deployment-related shame (Shame and Guilt After Trauma Scale [SGATS], Aakvaag et al., 2016) items and response options**

- *Worried about what people might think of you after what happened.*
- *Tried to conceal what happened, or any part of it.*
- *Felt ashamed about any part of what happened.*
- *Looked down on yourself after what happened.*
- *Blamed yourself for any part of what happened.*
- *Bothered by thoughts that you should have done something differently to prevent what happened.*
- *Bothered by thoughts that you should have done something differently while it was happening.*
- *Felt that you did anything wrong.*
- *Experienced any feelings of guilt about any part of what happened.*

With reference to traumatic experiences during deployment, the items are rated in terms of agreement using three response options: 1. No, 2. Yes a little, and 3. Yes a lot.

The original items were in Norwegian and published in English by Aakvaag et al. (2016).

**Anxiety and Depression (Hospital Anxiety and Depression Scale [HADS], Zigmond & Snaith, 1983) items and response options**

- *Feel tense or 'wound up'*
- *I feel as if I am slowed down.*
- *I still enjoy the things I used to enjoy.*
- *I get a sort of frightened feeling like 'butterflies' in the stomach.*
- *I get a sort of frightened feeling as if something awful is about to happen.*
- *I have lost interest in my appearance.*
- *I can laugh and see the funny side of things.*
- *I feel restless as I have to be on the move.*
- *Worrying thoughts go through my mind.*
- *I look forward with enjoyment to things.*
- *I feel cheerful.*
- *I get sudden feelings of panic.*
- *I can sit at ease and feel relaxed.*
- *I can enjoy a good book or radio or TV program.*

Items are rated on a scale from 0 to 4 with reference to the last week; 0 represents low intensity or frequency and 4 represents high intensity or frequency.

**Insomnia (Insomnia Severity Index [ISI], Bastien et al., 2001) items and response options**

- *Difficulty falling asleep. (None to Very)*
- *Difficulty staying asleep.* *(None to Very)*
- *Problem waking up too early in the morning.* *(None to Very)*
- *How satisfied/dissatisfied are you with your current sleep pattern? (Very Satisfied to Very Dissatisfied)*
- *To what extent do you consider your sleep problem to INTERFERE with your daily functioning (e.g. daytime fatigue, ability to function at work/daily chores, concentration, memory, mood, etc.) (Not at All Interfering to Very Much Interfering)*
- *How NOTICEABLE to others do you think your sleep problem is in terms of impairing the quality of your life? (Not at All Noticeable to Very Much Noticeable)*
- *How WORRIED/distressed are you about your current sleep problem? (Not at All to Very Much)*

Items are rated on a scale from 0 to 4 with reference to the last two weeks; 0 represents low problem levels and 4 represents high problem levels. Response ranges are included in parentheses after each item.

**Hazardous Drinking (Alcohol Use Disorder Identification Test [AUDIT], Babor et al., 2001) items and response options**

- *How often do you have a drink containing alcohol? (Never, Monthly or less, 2-4 times a month, 2-3 times a week, 4+ times a week)*
- *How many drinks containing alcohol do you have on a typical day when you are drinking? (1 or 2, 3 or 4, 5 or 6, 7 to 9, 10 or more)*
- *How often do you have six or more standard drinks on one occasion? (Never, Less than monthly, Monthly, Weekly, Daily or almost daily)*
- *How often during the last year have you found that you were not able to stop drinking once you had started? (Never, Less than monthly, Monthly, Weekly, Daily or almost daily)*
- *How often during the last year have you failed to do what was normally expected of you because of drinking? (Never, Less than monthly, Monthly, Weekly, Daily or almost daily)*
- *How often during the last year have you needed a drink first thing in the morning to get yourself going after a heavy drinking session? (Never, Less than monthly, Monthly, Weekly, Daily or almost daily)*
- *How often during the last year have you had a feeling of guilt or remorse after drinking? (Never, Less than monthly, Monthly, Weekly, Daily or almost daily)*
- *How often during the last year have you been unable to remember what happened the night before because of your drinking? (Never, Less than monthly, Monthly, Weekly, Daily or almost daily)*
- *Have you or someone else been injured because of your drinking? No, Yes, but not in the last year, Yes, during the last year)*
- *Has a relative, friend, doctor or other healthcare worker been concerned about your drinking or suggested you cut down? (No, Yes, but not in the last year, Yes, during the last year)*

Items 1 – 8 are scored as 0, 1, 2, 3 or 4, while items 9 and 10 are scored as 0, 2 or 4 only. All responses are summarized to create a total score. Response options are included in parentheses after each item.

**Satisfaction with life (Satisfaction with Life Scale [SWLS], Diener et al., 1985) items and response options**

- *In most ways my life is close to my ideal.*
- *The conditions of my life are excellent.*
- *I am satisfied with my life.*
- *So far I have gotten the important things I want in life.*
- *If I could live my life over, I would change almost nothing.*

Items are rated on a scale from 1 to 7 with reference to current perception of life; 0 represents strong disagreement with statement, 4 represents neither disagree nor agree with statement, and 7 represents strong agreement with statement.
